# Populism, “Anti” Ideologies, and Feminist Coalitions

**DOI:** 10.3389/fsoc.2020.620065

**Published:** 2021-02-17

**Authors:** Brooke A. Ackerly

**Affiliations:** Political Science Department, Vanderbilt University, Nashville, TN, United States

**Keywords:** philanthropy, feminism, activism, anti-racism, coalitions, social movements

## Abstract

By understanding populism as an “anti-” politics we can see two strands of populism: the *anti*-democratic strand which marginalizes certain groups of people and the anti-structural injustice strand coming from marginalized people. The potential of this anti-structural injustice activism encourages activists to expand their coalitional politics and government and philanthropic donors to see the import of funding and otherwise supporting work against structural injustice that explicitly takes on patriarchy and racism, among the full gamut of ideologies based on hierarchy and injustice.

Populism draws on ideology to mobilize outside of the constraints of politics. These constraints vary by context and might include party structure and international institutions. To mobilize without the support of the conventional political architectures in their context, populist leaders identify untapped ideological well-springs, often those that reify groups of people as the cause of the current negative conditions. Thus, despite significant differences among populist leaders from the US and Venezuela, Brazil and the United Kingdom, their similarities are alarming to feminists because regardless of against which groups they mobilize, these leaders have found patriarchy a bountiful ideological wellspring for mobilization. Likewise, feminist activists have mobilized outside the constraints of politics.^1^ Around the world working locally and intersectionally in partnership and across the world networked with anti-globalization, climate justice, anti-racism, immigrant, and decolonial movements feminist activists have been drawing on a different ideological well-spring. Their social justice ideological well-spring is different from the outgroup-focused ideologies of authoritarian populism because it is against structural injustice and because it commits its participants to pursue transformative change. To set up the argument, I follow Nadia Urbinati's analysis of the “anti-politics” *ideologies* of populists, in order to illustrate how the tools of populists can and in fact have been used by those offering non-authoritarian alternatives to existing politics. Next, I focus on the structural obstacles to the strand of anti-structural injustice feminist populism that I identify. I conclude, perhaps surprisingly given concerns feminists have raised about the transformative potential of philanthropy, with arguments about how the established mechanisms of mobilizing resources outside of democratic politics can be used to support a politics that resists structural injustice.

The contributors to this set of Perspectives on populism come together around the puzzle that “populist movements often appeal to gender, sexual, racial, disability, class, and national stereotypes in fostering distrust of democratic political institutions, even as they profess to support the empowerment of “the people” (Botting and Gould this issue). Both for understanding these anti-democratic movements and for identifying the political resources for democratic responses, we need to focus more on the ideologies that these leaders access than the charisma with which they mobilize them. There are many charismatic people, but they don't all mobilize anti-democratic, anti-intellectual, or anti-elite movements. Rather, citing Rosanvallon (2006), Nadia Urbinati argues that populism is a “negative politics” (Urbinati, 2019, p. 113): “[P]opulism's adversarial identity is claimed by a representative leader, who mobilizes the media to convince the audience that he embodies the people's many forms of discontent against traditional parties' spineless mainstreamism” (Urbinati, 2019, p. 113). This framing makes it contextually versatile and potentially dangerous, but as I argue, it is also potentially fruitful for recognizing the cross-coalition mobilizations of critics of structural injustice as a populist anti-politics as well.

Anti-politics is dangerous to democracy when leaders mobilize this politics of negatives against particular groups. As many have noted, it relies on anti-intellectualism and anti-elitism neither of which is particularly ideological or antidemocratic. Populists becomes nefariously anti-democratic when they mobilize ideologies such as patriarchy, racism, caste, and xenophobia. Such hierarchical ideologies provide a well-spring for directing dissatisfaction with current conditions toward resentment of groups of certain people rather than at the injustice itself that is the true cause of those conditions. Thus, the support for populist leaders need not depend on their ability to change the conditions.

By contrast popular movements against structural injustices likewise draw on ideological well-springs, but theirs are committed to changing structural conditions; they draw on ideologies that unite the people against oppressive political, economic, and social structures. Their differences include those who are more reform minded and those guided by a revolutionary vision. A decade of global activism that drew on local activism and culminated in the Beijing Declaration and Platform for Action of the 1995 UN Conference for women, a platform that supported equality, development, and peace, at the turn of the millennium. However, within the next decade feminists around the world felt under attack from all directions. Misogyny took many forms and seemed integrated into patriarchy, neo-imperial wars, conservative religious fundamentalism, militarism, and neoliberal economic politics (particularly those free-trade policies that favored capitalists over workers, relied on unpaid reproductive work, and utilized the environment and natural resources with minimal attention to sustainability).

Any debate among feminists (activists and academics) about whether reform or revolution was the right strategy was overshadowed by backlash. In the United Nations-sponsored conferences after Beijing, coalitions between religious fundamentalists and capital-centric economic “fundamentalists” threatened to rollback achievements of the global women's movement of the previous decade and to undermine the ability of local activists to use them. Additionally, domestic political and cultural leaders threatened local activists with accusations that their feminism was a culturally foreign influence and not a direct response to the injustices people in their communities faced (Rothschild et al., 2005). With their own countries and globally, feminists responded both by reaching out to possible allies in, and by being a part of, the anti-globalization movements, the World Social Forum movements, labor movements, anti-war movements, immigrant movements, environmental justice movements, and domestic worker movements. Some of these networks were based in identity politics, with multiply-identified, often queer women of color being the bridges and translators (Mayo-Adam, 2020). Some networks were coalitions across difference. Yet, generally intersectional analysis enabled both identity-based and strategy-based networking and alliance building. Intersectional analysis enables feminists to identify ways in which structural oppressions are imbricated. Feminist activists, locally and through transnational networks, build partnerships and alliances so that transformations in any terrain of structural conditions strengthen the possibilities and options for further transformations (Ackerly, 2001, 2008, 2018).

Women considered making the World Social Forum a site for their activism and met together in 2004 and 2005 in Feminist Dialogues before the World Social Forum to determine how to work within and with the forum of movements of movements (Hewitt, 2008). They alighted on a trinity of resistances: anti-militarism, anti-fundamentalisms (which included anti-neoliberal economic fundamentalism), and anti-patriarchy (Feminist Dialogues (George and Soaki, 2020). These commitments “made up of negatives” (to use Urbinati's phrase) were and continue to be well-spring ideologies against structural injustice that feminists share with many social movements.

In practice, feminists have drawn on these ideologies to unite across issues such as domestic worker rights (e.g., International Labor Organization., 2011), gender justice in climate change (Terry, 2009), and anti-racism (Twine and Blee, 2001). Like anti-democratic populism, feminist popular movements draw on an ideology of resistance. The difference is that while the anti-democratic populists create common ground against certain groups of people, feminist populism creates common ground against structural oppressions.

Given what amounts to (at least) two decades of feminists' allied anti-structural injustice activism, why isn't it more of a political force?

Well, first, we might reject the premise. There is significant evidence that anti-structural injustice feminist-informed activism is a significant force. Cross-national comparisons of policy transformation (Htun and Weldon, 2010, 2018; Weldon and Htun, 2013) and political repression of feminist activism and scholarship are two kinds of evidence of its effectiveness (Demirtaş and Gündüz, 2020; O'sullivan and Krulišová, 2020). Non-feminist actors taking over feminist goals is also evidence of success. For example, in Cyprus, unions and to some extent political parties incorporated the goals of feminist movements such that the feminism behind these became invisible (Kamenou, 2020, p. 372). Achievements such as ILO 189, the Convention on Domestic Workers, are evidence of success as is the mobilization of protestors who come out together across intersecting issue areas (e.g., Women's March 2017 and Women Radically Transforming a World in Crisis 2019^2^). In fact, the thesis of a recent popular press book is that an entire generation of US college students have fully adopted the view that all harms are a form of structural injustice (Lukianoff and Haidt, 2018).

Despite evidence that feminist anti-structural injustice coalitional mobilization has been effective in some contexts, to the extent feminist-infused anti-structural injustice movements are not as strong as the ideas that support them, what might explain that? It could be that these movements don't hold together. There may be examples of them holding together and others of them not holding together, but there seems to be evidence that attending to structural injustice doesn't divide movements; it strengthens them, particularly when women are central actors (Ackerly, 2018) and when women mobilize locally in their own vernacular, with their own “spin,” in their everyday “little nothings” for transformative change (Drumond and Rebelo, 2020; Singh, 2020).

Another possibility is that while broad coalitional movements can bring attention to issues, getting things done is a grassroots enterprise and we all cannot do everything. Thus, the practical divisions of labor that enable people to get things done may result in their not being as coalitional in their day to day work, even if they are coalitional in their analysis of their problems and share an ideology of anti-structural injustice. For example, the Platform for Action is organized into 12 critical areas of concern. While these overlap, and while work on them should overlap, the disaggregation that may be practically necessary has a political consequence that may include undermining of networking and collaboration across issues.

Further, it could be that they rely on government policy or government and philanthropic funding and thus by some ideologies cannot be revolutionary at their core (Incite! Women of Color against Violence., 2007). At the same time that funding for these activities has increased in some places on some issues, there has also been an emphasis on measurement and efficacy (Teles and Schmitt, 2011). Dominant interpretations of what constitute legitimate measurements of achievements and efficacy has resulted in more funding for initiatives that are able to articulate clear measurable goals within a specified timeframe. Goals related to political transformation of unjust social, economic, and political structures are not well-captured in these measurement frameworks.

Most critically, it could be that they rely on funding but that government and philanthropic funding does not support political movements. Incite! argues that the criminal justice system undermines women's ability to organize (Incite!, 2001) and more generally that what is needed is a revolution and a revolution will not be funded (Incite! Women of Color against Violence., 2007).

However, before rejecting the role of philanthropy in potentially transformative politics, it is worth looking at cases in which it has worked. Women's movement organizations have decades (in fact we are celebrating over a century) of experience working against the full gamut of oppressive politics. Their movements and movement organizations are as varied as women and their contexts. Women's funds are relatively small philanthropic organizations that accept donations and make grants in relatively small amounts to women's organizations. Examples include the Global Fund for Women, MamaCash, Urgent Action Fund, Astraea, Frida, and the networked members of Prospera. Feminist philanthropy takes on power dynamics even though the sources of philanthropic resources may be deeply implicated in these. By looking at the work that women's funds support, we can see that a process exists for supporting anti-structural injustice movements that resist “anti” group discourses and undermine “othering” but which, like antidemocratic populism, are able to form coalitional politics across concerns about structural injustice (Ackerly, 2018).

Across contexts, when the ideological well-springs against injustice exist, but face political obstacles to building alliances and networks, philanthropic support can facilitate movement building by creating alternative spaces. These can be global, local, or both. Moreover, they can include alliances among those with more revolutionary aspirations and those who are more grounded in reform-oriented strategies. Regardless of revolutionary or reform orientation, anti-structural injustice activists are seeking to rebuild the social, economic, and political metaphorical ship of society and both acknowledge that the ship is at sea. In the activist realm, the revolution/reform distinction can be important in ideological orientation, but semantic in practice.

To conclude, in Urbinati's review of populism, there is an important caution for democracy: populist leaders can ride a wave of support to a level of political power that is no longer able to be constrained by democracy. This risk suggests that those governments and philanthropic donors who have supported the criticism of patriarchy need to revisit their willingness and ability to fund the coalitional politics that it requires. As we turn our attention to anti-democratic populism, and the attention it garners, we can also draw attention to the coalitional politics that have characterized feminist movements globally. Recognizing that not always, but everywhere feminists have been leading and part of coalitional and networked politics that provide an ideological alternative to the anti-democratic well-spring of the outgroup politics of populists, we might consider learning more about where the funded-revolution is taking hold and able to resist anti-democratic populism by fostering an inclusive political alternative.

In brief, we make a mistake when we center anti-democratic populism in our discussions of populism. Rather, we should recognize the political tools attributed to populists and their leaders and more importantly, identify the political tools that can create another world.

## Author Contributions

The author confirms being the sole contributor of this work and has approved it for publication.

## Conflict of Interest

The author declares that the research was conducted in the absence of any commercial or financial relationships that could be construed as a potential conflict of interest.

## Footnotes

^1^Feminists have also mobilized directly against nationalist populism (Kamenou, 2020).

^2^A framework for Beijing+25 shaped at a strategy meeting of feminist activists. “The government of Mexico invited a group of feminist experts to advise them on the content and format of the Generation Equality Forum and the Mexico City launch. Participants included: Articulación Feminista Marcosur; Asia Dalit Rights Forum; Asia Pacific Forum on Women, Law and Development; Association for Women's Rights in Development; Center for Women's Global Leadership; CatchAFyah; Development Alternatives with Women for a New Era; Diverse Voices and Action for Equality; Fundación Arcoiris por el respeto a la diversidad sexual; Fundación para el Estudio e Investigación de la Mujer; Global Fund for Women; Huairou Commission; Instituto da Mulher Negra; International Center for Research on Women; International Forum of Indigenous Women; International Women's Health Coalition; Just Associates; Las Reinas Chulas; Luchadoras; Mexico and Central American Women's Human Rights Defenders Initiative; Musas de Metal; Nazra for Feminist Studies; Realizing Sexual and Reproductive Justice; Regions Refocus; Shibuye Community Health Workers; Shirkat Gah-Women's Resource Centre; Spectra; University of São Paulo; and Women in Migration Network (http://bit.ly/B25Framework).
